# Editorial Department

**Published:** 1884-07

**Authors:** 


### The Bistoury.

ELMIRA, N. Y., JULY, 1884.

The BISTOURY is published Quarterly, upon the first of April, July, October and January, at Fifty Cents a year in advance.

### Editorial Department.

BOTH SIDES.—The articles by Dr. Carr in the April and July numbers of the BISTOURY, are ably answered by Dr. Blackham, explaining the fallacies of Dr. Carr's line of argument. It is very well, we think, to ventilate both the error and its antidote, such a course being calculated to correct popular mistakes.

DANGEROUS TO LIVE.—Our friend H—— says, in view of recent developments in science, that "it is becoming dangerous to try to live." M. Parize, a French savant, has been making microscopical examinations of the interior of bricks and brick walls generally and finds them full of the ubiquitous bacterium, which proves to be the germ of so many obscure diseases. We have not heard from H—— yet on this.

AGENTS FOR MEDICAL HOUSES.—A call from these gentlemen is wrongfully considered a nuisance by some physicians. We have found such visits frequently not only agreeable but instructive. These agents are generally men of intelligence and are thoroughly posted on the specialties of the manufacturers whom they represent. The careful and attractive manner in which these officinal preparations are now compounded and put up is winning the confidence of the profession and saving patients from incompetent pharmacists. Give these gentlemen welcome, and you will keep up with the times whilst enjoying their visit.

CHEAP EXCURSIONS.—Interest and convenience seem to go hand in hand in many instances in the larger cities. Men of capital combine to make attractive neighboring places of resort, and railroad and steamboat companies soon find it their interest to furnish the means of access at exceedingly low rates, thus the convenience, comfort and pleasure of the public are served at prices within the reach of all. From New York to Coney Island with its attractions, both natural and artificial, the fare is only twenty-five cents for the round trip, with a choice of railroad or steamboat either way. From Philadelphia to Cape May, ninety-six miles, an elegant steamboat, the "Republic," runs the round trip daily for one dollar. Other instances might be cited and indeed we are glad to note that cheap excursions are becoming general throughout the country and are being patronized by a class of persons who will add to their popularity.

A BAD HABIT.—There is a habit prevailing amongst travelers, and we are sorry to say most frequently the culprit is a female, of opening car windows without regard to the state of the weather or the feelings of those who are to be incommoded, and perhaps made sick by the cold air introduced. A person who has been hurrying to a train or who is otherwise excited enters a car where the other inmates have become accustomed to the atmosphere and to whom the ventilators, above, are ample, and in order to hasten her own accomodation to the change of air incommodes every one in her immediate vicinity by insisting on keeping a window open. We would suggest that a rule might be made to cover this and suit all parties. The conductor might offer the offender the choice of a back seat or the alternative of being contented with the ventilation which has met the wishes of the other occupants of the car.

SUMMER MUNIFICENCE.—Upon a recent visit we were struck with the lavish expenditure of those residents of New York who have their summer homes on Long Island; magnificent cottages which make one wonder what kind of a city palace the owners occupy in winter. Of course the style of living is equally extravagant—immense retinues of servants, valuable stables of horses, Tally Ho coaches with gentlemen drivers and outriders meeting the trains and escorting friends and neighbors home—all examples of the easy way which money comes and goes in the great metropolis. At Babylon is an elegant hotel, the "Argyle," owned and run by "The London American Improvement Co.," of which the Duke of Argyle's son is president. The hotel has attached to it about twenty elegant cottages which rent from eight hundred dollars upward, the cottagers having table board at the hotel at eight dollars per day.

TRADE ENTERPRISE.—The secret of successful advertising seems to be more generally understood and its results appreciated now than ever before. It is curious to notice the many devices involving great outlays of money, which are resorted to. Standing in some very prominent field may be seen an immense elephant carved out or built up and bearing the trade mark or sign of the enterprising advertiser. Again, a "counterfeit presentment" of a train of cars with a locomotive attached, attracting the passer to read the legend of some popular clothing or dry goods house. The whole page of some of the most expensive advertising sheets will be taken at great cost to draw attention to some live merchant's stock. All this must pay in its result or it would not be done. There is scarcely a successful house in the country, which does not owe its prominence to judicious advertising. Perhaps this may lead others to a trial and success.

MIRACULOUS CURES.—How much faith has to do with many cures which are wrought by simple remedies under the direction of old Indians, gypsies, grannies and even M. D.s we shall probably never know. Nor what real efficacy there is in the money spent at certain shrines as Lourdes and others, but we do know that the temptation for deception is very great and that it is quite as good fun for the deceiver as it is for the deceived, especially as the weight of profit is on the side of the former. We have sometimes thought that persons are to be found who actually deceive themselves into even the most absurd belief. We met with a man some years ago who had told the following so frequently that it was thought he really believed it: He was one day in the woods and sat down on a root to rest, leaning his back against the stump. After a few minutes he was conscious of a tickling sensation on his ear. He tried to brush away the intruding insect with his hand; but upon a repetition of the attack he looked around and discovered a rattlesnake tickling his ear. The difficulty was that he was sitting on the snake's tail! The probabilities of the truth of this story are about equal to those of the wonderful miraculous cures claimed for the waters and Charlatans referred to.

CONTAGIOUS DISEASES.—Professor Tyndall says that the rarity of second attacks of communicable diseases is due to the removal from the system by the first parasitic crop, of some ingredient necessary to the growth and propagation of the parasite.

It is to be hoped that some substance will be found which will destroy the contagion and thus prevent the multiplication in the blood and tissues of these micro-organisms which are so destructive to the constitution. Scientists are busy at work trying to find some effectual means of rendering the body invulnerable against contagious diseases, and to a slight extent they have been successful.

About the only way immunity from this class of diseases can be obtained is by isolation, cleanliness and thorough disinfection of the substances propagating the malady, rendering them incapable of supporting microscopic life and ending in their complete effacement.

Too much attention can not be expended by our councilmen to rid the city of any foul matter which would contribute a defiling influence to its surroundings, money used for the promotion of cleanliness is well appropriated.

A REVIVAL NEEDED—Audiences in this country are proverbially forbearing, and not unless the provocation is very excessive will they display their disapproval save only by strict silence. Now this is not good for either the performers or the listeners. It is likely to, and does lead to a kind of apathy on both sides which is not provocative of exertion to please or calculated to develop much pleasure. When a magnetic sympathy is once established between the singer and his audience, when he is sure of the manifested approval of his successful rendition of the role, then he can and will give his best efforts, and his manager will be careful to cast him in nothing beyond his reach. Especially will that be the case if it is understood perfectly that whilst success will be encouraged and rewarded, the slovenly rendition will as certainly be condemned. An Italian audience will manifest its displeasure immediately at a false note by a groan. Actors and actresses know too well the cost of most newspaper praise or forbearance, and in some instances prefer to pay rather than play. Let us revive again the privilege of the audience of doing its own criticizing, clap when pleased and hiss when displeased. The price of admission now demanded should entitle the payer to the services of actors capable of properly filling the parts for which they are cast.

